# Environmental enrichment may protect against hippocampal atrophy in the chronic stages of traumatic brain injury

**DOI:** 10.3389/fnhum.2013.00506

**Published:** 2013-09-24

**Authors:** Lesley S. Miller, Brenda Colella, David Mikulis, Jerome Maller, Robin E. A. Green

**Affiliations:** ^1^Applied Psychology and Human Development, Ontario Institute for Studies in Education, University of TorontoToronto, ON, Canada; ^2^Cognitive Neurorehabilitation Sciences Lab, Research Department, Toronto Rehab-University Health NetworkToronto, ON, Canada; ^3^fMRI Laboratory, Division of Applied and Interventional Research, Toronto Western Research Institute, University Health NetworkToronto, ON, Canada; ^4^Faculty of Medicine, Department of Medical Imaging, University of TorontoToronto, ON, Canada; ^5^Brain Stimulation and Neuroimaging Laboratory, Monash Alfred Psychiatry Research Centre, Alfred HospitalMelbourne, VIC, Australia; ^6^Graduate Department of Rehabilitation Science, University of TorontoToronto, ON, Canada

**Keywords:** traumatic brain injury, environmental enrichment, subacute atrophy, adult, moderate to severe

## Abstract

**Objective:** To examine the relationship between environmental enrichment (EE) and hippocampal atrophy in the chronic stages of moderate to severe traumatic brain injury (TBI).

**Design:** Retrospective analysis of prospectively collected data; observational, within-subjects.

**Participants:** Patients (*N* = 25) with moderate to severe TBI.

**Measures:** Primary predictors: (1) An aggregate of self-report rating of EE (comprising hours of cognitive, physical, and social activities) at 5 months post-injury; (2) pre-injury years of education as a proxy for pre-morbid EE (or cognitive reserve). Primary outcome: bilateral hippocampal volume change from 5 to 28 months post-injury.

**Results:** As predicted, self-reported EE was significantly negatively correlated with bilateral hippocampal atrophy (*p* < 0.05), with greater EE associated with less atrophy from 5 to 28 months. Contrary to prediction, years of education (a proxy for cognitive reserve) was not significantly associated with atrophy.

**Conclusion:** Post-injury EE may serve as a buffer against hippocampal atrophy in the chronic stages of moderate-severe TBI. Clinical application of EE should be considered for optimal maintenance of neurological functioning in the chronic stages of moderate-severe TBI.

## Introduction

Conventionally, moderate-severe traumatic brain injury (TBI) has been viewed as a non-progressive brain disorder with a predictable trajectory of recovery leading to a stable course thereafter (Lezak et al., 2004). However, growing research findings show progressive gray matter atrophy and loss of white matter integrity in the post-acute and chronic phases of injury (Trivedi et al., 2007; Greenberg et al., 2008; Ng et al., 2008; Farbota et al., 2012; Adnan et al., in press). Further, some individuals with TBI also show progressive cognitive and functional declines in the ensuing months and years following injury (Corkin et al., 1989; Millis et al., 2001; Himanen et al., 2006; Till et al., 2008), supporting the notion that TBI is not a stable condition (Ng et al., 2008; Till et al., 2008).

A brain structure of key importance in TBI is the hippocampus. Memory impairment is one of the most common complaints following TBI, in part due to the acute effects of hippocampal injury, which include excitotoxic and hypoxic insult (Rosenfeld et al., 2012). On top of these acute injuries, post-acute atrophy of the hippocampus has now been demonstrated in several TBI studies (Bigler, 1997; Ng et al., 2008). Tate and Bigler (2000) have suggested that post-acutely, hippocampal cell loss may be the result of transneuronal degeneration secondary to hippocampal deafferentation and/or deefferentation. Indeed, the unique pattern of neuronal projections within the hippocampus (Duvernoy et al., 2005), which is comprised of six architecturally distinct regions linked via unidirectional projections (Amaral and Witter, 1995), has been referred to as an Achilles' heel of sorts, whereby damage to one region of the hippocampus can lead to downstream damage to other regions via loss of activity-dependent survival factors (McCarthy, 2003). Thus, disuse-mediated loss, either secondary to disconnection or to a dearth of behavioral stimulation, may be an important factor in observed hippocampal volume loss in the post-acute stages of injury.

Disuse-mediated loss—or “use it or lose it”—is a concept that has received extensive attention in the older adult literature. It has been argued for decades that greater day-to-day cognitive stimulation is associated with less cognitive decline and delayed onset of dementia. The “negative neuroplasticity” framework of Mahncke et al. (2006), advanced to explain functional losses in normal aging, offers a disuse-mediated framework that can also be applied to atrophy in the chronic stages of TBI (Evans et al., 2008). In their framework, the authors posit that cognitive, perceptual, emotional, physical, psychosocial, and vocational changes associated with aging limit engagement in the busy schedules and complexity of activities of earlier life, leading to a cycle of behavioral inactivation and avoidance—or learned disuse (Taub et al., 2006), and resulting in reduced activation of brain networks (Blake et al., 2006). Although behavioral losses are more sudden in brain injury, moderate-severe TBI patients, too, are commonly unable to engage in the complex activities of work, school and social activities as a result of TBI-induced decrements in cognitive, physical, perceptual and emotional functioning. These patients are especially at risk of reduced behavioral (and thereby neural) stimulation in the sub-acute and chronic stages of injury (after discharge from in-patient rehabilitation facilities), where there is often reduced environmental stimulation and/or reduced supports to foster engagement in the environment (Evans et al., 2008; Frasca et al., 2013).

Related to the above, the concept of environmental enrichment (EE) refers to exposure to and engagement with complex and stimulating environments, and there is extensive evidence that EE can beneficially influence the size, morphology and function of the brain, through synaptic modification and synaptogenesis, the shape and size of dendritic spines (or spine remodeling), axon collateral sprouting, hippocampal (dentate gyrus) neurogenesis and survival of neurons, and brain network connectivity; and such changes have been associated with improvements in functional performance (Rosenzweig and Bennett, 1996; Diamond, 2001; Mohammed et al., 2002; Draganski et al., 2006; Kasai et al., 2010; Kolb et al., 2010; Berlucchi, 2011). Indeed, seminal reviews of the literature conclude that brains that have received increased stimulation, via enhanced mental and physical activity, are better able to mount neuroprotective responses against neurodegenerative processes, traumatic insults and other forms of adult-onset neural dysfunction (Nithianantharajah and Hannan, 2009).

Taken together, the above literature (Mohammed et al., 2002; Kolb et al., 2010; Berlucchi, 2011) suggests that environmental influences, such as intensified cognitive stimulation, might buffer against chronic stage atrophy in moderate-severe TBI. The primary objective of the present study, therefore, was to examine the relationship between post-injury EE, and hippocampal atrophy in the chronic stages of injury. To our knowledge, no studies have yet examined this relationship. We hypothesized that greater EE (i.e., frequency of cognitive, physical, and social engagement) measured in the early post-acute stages of injury (i.e., 5 months post-injury) would be associated with less long-term bilateral hippocampal atrophy over the ensuing years (measured from 5 to 28 months post-injury).

The secondary objective concerned pre-injury EE factors, namely exposure to higher levels of education in development and early adulthood, which has been associated with better cognitive recovery following TBI (Kesler et al., 2003). Given evidence of (1) a positive association between education and cognitive and functional recovery following TBI (Green et al., 2008) and stroke (Elkins et al., 2006), (2) a positive association between education and dendritic branching in healthy adult humans (Jacobs et al., 1993), and (3) a positive correlation between years of education and cognitive functioning in normal aging (Corral et al., 2006; Fritsch et al., 2007), it was hypothesized that greater years of education would be associated with less bilateral hippocampal atrophy, measured from 5 to 28 months post-injury.

## Methods

### Participants

The 25 clinical participants in this study (see Table 1) were part of a larger research study being conducted at the Toronto Rehabilitation Institute, a large publicly-funded inpatient neurorehabilitation hospital. The focus of the larger study was to investigate the natural history and mechanisms of recovery following moderate-severe TBI. Informed consent was obtained from all participants in the study, and procedures for the present study were approved by the Research Ethics Board of the Toronto Rehabilitation Institute and the Office of Research Ethics at the University of Toronto. Participants underwent prospective assessments at approximately 5, 12, and 28 months post-injury. As the current design was a retrospective analysis of data collected in the course of the larger study data were *only* available at the 5, 12, and 28 month time points.

**Table 1:** **Demographic information and hippocampal volume loss**.

**Subject ID**	**Age/sex**	**Education**	**GCS**	**PTA**	**IQ (WTAR)**	**% Change in bilateral hippocampal volume from 5–28 months post-injury**
368	68/M	8	Unknown	5	ESL	−19.79
380	48/M	12	3	4	89	−14.45
358	59/F	17	4	5	107	−14.27
376	29/M	16	3	4	124	−11.06
364	54/M	20	7	5	120	−8.82
369	25/M	12	Unknown	5	LD	−7.91
362	60/M	15	Unknown	5	111	−7.05
337	17/M	12	4	1	83	−6.85
363	46/M	17	3	5	Dysarthria	−6.01
389	48/M	11	6	6	105	−5.49
353	27/F	18	3	5	117	−5.35
352	59/M	12	3	6	104	−4.79
339	37/M	12	3	6	89	−4.46
333	44/F	16	6	4	120	−4.06
379	31/M	16	8	6	108	−3.31
349	50/F	17	10	3	120	−3.29
338	57/F	12	13	6	98	−3.27
359	18/F	12	6	5	LD	−2.55
329	19/M	9	Unknown	4	103	−0.97
346	28/F	14	7	5	106	0.27
374	66/M	12	6	5	Aphasia	0.83
331	30/M	16	6	6	97	2.77
327	41/M	11	3	4	99	2.98
330	40/F	13	3	0	116	3.34
335	25/M	16	7	4	124	5.43
	(*M* = 41yrs; *SD* = 16)	(*M* = 14 yrs; *SD* = 3)	(*M* = 5; *SD* = 3)	(*M* = 4.6days; *SD* = 1.5)	*(M =109; SD =11.4)*	(*M* = −4.7%; *SD* = 6.0)

Inclusion criteria for the larger study comprised: (1) acute care medical diagnosis of TBI; (2) posttraumatic amnesia (PTA) of 1 h or more and/or Glasgow Coma Scale (GCS) score of 12 or less either at emergency or at the scene of the accident and/or positive CT or MRI findings; (3) age between 17 and 80 years old; (4) able to follow simple commands in English based upon Speech Language Pathologist intake assessment; and, (5) competent to provide informed consent for study or availability of a legal decision maker.

Exclusion criteria for the larger study included: (1) orthopaedic injuries affecting both upper extremities; (2) diseases primarily or frequently affecting the central nervous system, including dementia of Alzheimer's type, Parkinson's disease, multiple sclerosis, Huntington's disease, lupus, or stroke; (3) a history of psychotic disorder; (4) non-emergence from posttraumatic amnesia by 6 weeks post-injury, as measured by the Galveston Orientation Amnesia Test (Levin et al., 1979); (5) TBI secondary to another neurological event, such as a fall due to stroke; and, (6) failure on a symptom validity test (Test of Memory Malingering) (Tombaugh, 1996) at any of the assessments. The demographic and injury characteristics of the clinical sample are shown in Table 1. The additional inclusion criteria for the current study were: relevant behavioral and MRI outcome measures at 5 months post-injury plus MRI data at 28 months post-injury.

There were 30 individuals from the larger study with all relevant outcome measures at 5 months post-injury who had reached or surpassed the 28 month post-injury time-point at the time of data analysis. Of those, 25 underwent the 28-month post-injury MRI and were, therefore, eligible for inclusion in the present study, indicating a retention rate of 83%.

Of the sample included in the present study, none showed evidence of severe anxiety or depression, and less than 30% reported experiencing mild or moderate depression or anxiety during the first year post-injury based on the Beck Depression Inventory (Beck, 1987) and Beck Anxiety Inventory (Beck, 1990).

### Materials

#### Lifestyle activities questionnaire

Assessment of post-injury EE was based on participants' self-reported frequency of engagement in a variety of activities involving cognitive, physical, and social demands. Given the absence of reliable or valid published measures of EE in humans at the time of data analysis, EE activities were listed on a self-report questionnaire designed for the purpose of the present study, entitled the Lifestyle Activities Questionnaire (LAQ). Cognitive, physical, and social activities on the LAQ were obtained from a theoretically-derived and empirically-tested inventory developed by Salthouse et al. (2002), which was constructed by specifying 22 common activities that a sample of 1200 adults, ranging from the age of 18–97 years, rated in terms of their cognitive demand (where 1 = low demand, corresponding to sleeping, and 5 = high demand, corresponding to working on a tax form). An activity added to the LAQ that was not on Salthouse's inventory was the frequency of engagement in sports or physical activity at the gym. Participants were asked to rate the frequency with which they engaged in each of the listed activities on an ordinal scale ranging from 1 to 5.

The scale comprised descriptors that ranged from (1) *didn't do at all* to (5) *several hours, every day of the week*. For statistical analysis, each number of the scale was weighted based on estimated associated hours. “Didn't do at all and less than once a week” was assigned a weight of zero; “once or twice a week” was assigned a weight of 1 (i.e., for 1 h/week); “several times a week” was assigned a weight of 3 (3 h/week); “an hour or so most days” was assigned a weight of 7; and “several hours a day” was assigned a weight of 20 (20 h/week). Transformation of ordinal scores into weighted scale scores corresponding to estimated hours/week provided a meaningful and more ecological marker of EE. However, for patient responses, the scale was kept as ordinal (i.e., 1–5), because self-reported ratings were deemed by clinical experts consulted to be more easily understood by patients and less likely to result in missing or inaccurate data. We suggest that this approach enabled us to provide richer data than previous methods of aggregating EE activities, in which individuals were classified as “active” if they endorsed doing even one mentally-challenging activity for 1 h per week or more, for example (Bosma et al., 2002; Richards et al., 2003).

Items were sub-classified into cognitive, social or physical activity types to allow for a comparison of the respective influences of each on hippocampal volume loss. For all items, consensus was reached by two trained clinicians regarding assignment to a cognitive, social or physical aggregate. Nineteen items were classified as cognitive activities, 8 items were classified as social activities, and 2 items were classified as physical activities. For each subject, the weighted score for each item type (i.e., social, cognitive, physical) was summed to create a sub-aggregate score for each component of EE; the grand sum was the total score for the larger EE aggregate.

#### MRI acquisition protocol

MRI scans were acquired on a General Electric (GE) Signa-Echospeed 1.5 Tesla HD scanner (SIGNA EXCITE, GE Healthcare, Milwaukee WI), using an eight channel head coil. Sequences included sagittal T1 (TR/TE = 300/13 ms), slice thickness = 5 mm, space 2.5 mm, matrix 256 × 128 axial gradient recalled echo TR/TE = 450/20, flip angle = 20°, slice thickness = 3 mm no gap, matrix 256 × 192 axial fluid-attenuated-inversion-recovery TR/TE = 9000/45 ms, TI (inversion time) = 2200 ms, slice thickness = 5 mm no gap, matrix 256 × 192 axial fast spin echo proton density (PD)/T2 TR/TE 5500/30, 90 ms, slice thickness = 3 mm no gap, matrix 256 × 192. All above mentioned sequences were obtained with a 22 cm field of view. The high-resolution isotropic T1 weighted, three-dimensional IR prepped radio-frequency spoiled-gradient recalled-echo images TI/TR/TE = 12/300/5, TI, FA = 20, slice thickness = 1 mm no gap, matrix = 256 × 256 were acquired in the axial plane utilizing a 25 cm field of view. The entire scanning session lasted ~55 min.

#### Image processing and analysis

The MR images were transferred to a workstation for image processing. The scans were received in the Digital Imaging and Communications in Medicine file format and were subsequently converted into (Medical Imaging Network Common Data Form) file format that was created at McConnell Brain Imaging Centre of the Montreal Neurological Institute. Following this procedure, the files were anonymized.

A number of image processing steps were performed in order to make the MRI data usable for image analysis. First, an intensity non-uniformity correction was performed, followed by linear registration of the images into stereotaxic coordinates based on the Talairach atlas. The linear registration to Talairach coordinates was accomplished through 3D cross-correlation between a given volume and an average MR brain image previously converted into the Talairach coordinate system allowing for direct anatomical comparisons between subjects. Finally, a second non-uniformity correction was performed after the registration, which helped to remove any residual non-uniformity artifacts.

The hippocampi were manually outlined using Analyze 7.0 (Brain Imaging Resource, Mayo Clinic, MN) by an experienced tracer (JM) from coronally orientated MR images in the anterior-posterior direction. Calculations of volumes were computed automatically by multiplying the number of voxels traced in each slice, by their depth (i.e., slice thickness). As described by Watson et al. (1992, 1997), the anterior tip of the hippocampus until the slice before the opening of the crux of the fornix was measured as the hippocampal head and body and included the subiculum, CA1-(4) areas, and dentate gyrus. The hippocampus tail was measured from the slice immediately posterior to that which represented the last slice according to the Watson protocol (Watson et al., 1992, 1997) (see Maller et al., 2007 for a more detailed description of this procedure).

All raw hippocampal volumes were expressed in mm^3^. For the purpose of the present study, hippocampal volume change between 5 months (T1) post-injury and 28 months (T2) post-injury was measured using the following formula:

$$\text{Hippocampal volume change}=\frac{\left(\text{Vol T2}-\text{Vol T1}\right)}{(\text{Vol T2}+\text{Vol T1})\text{​}/2}*100$$

### Design and procedures

The study employed a retrospective, within subjects, longitudinal design. For clinical participants, LAQs were collected and initial MRI scans were acquired at a mean of 5.3 months (*SD* = 1.2; range = 4.3–10.3 months) post-injury; follow-up MRI scans were acquired at 28.48 months (*SD* = 5.5; range = 24–42 months) post-injury.

The primary dependent measure for the study was bilateral hippocampal volume. The primary independent measures were (1) EE as measured by the total score of the LAQ at 5 months post-injury and (2) years of education prior to the injury. We also examined the relative contributions of the cognitive, social and physical EE sub-aggregate scores.

The primary control variables were injury severity (measured by the Glasgow Coma Scale, duration of PTA, and duration of acute care length of stay), estimated premorbid intelligence (based on the mean score on the Wechsler Test of Adult Reading administered [Wechsler, 2001] at 12 and 28 months post-injury), socioeconomic status, and age at injury.

## Results

Using Pearson product moment correlation, primary control variables were correlated with hippocampal atrophy and with the measure of overall EE. Each variable was correlated separately due to limited power secondary to sample size. Any variables with significant correlation were included in subsequent analyses. No significant correlations were observed between any of the primary control variables and EE or hippocampal atrophy, with the exception of age, which correlated significantly with the EE aggregate (*r* = −0.45, *p* < 0.05, *N* = 25) whereby increasing age was associated with less overall EE. As well, there was a trend for higher age at injury to be associated with greater bilateral hippocampal atrophy (*r* = 0.39, *p* = 0.06, *N* = 25). These findings, combined with previous research findings of greater deleterious effects of injury on aging brains (Popa-Wagner et al., 2007; Onyszchuk et al., 2008; Petcu et al., 2008), necessitated controlling for age at injury in the present study analyses.

Partial correlation was used to test the primary hypothesis of the study, namely the relationship between the EE aggregate and bilateral hippocampal atrophy, while controlling for age at injury. A significant negative correlation was observed (*r* = −0.42, *p* < 0.05, *df* = 21) whereby greater general activity level at 5 months post-injury was associated with less bilateral hippocampal atrophy from 5 to 28 months post-injury.

Partial correlation between years of education and hippocampal atrophy, controlling for age at injury was used to examine the secondary hypothesis. Contrary to prediction, no significant relationship was observed (*r* = −0.05, *p* = 0.82, *df* = 22).

Lastly, we also explored preliminarily the respective contribution to hippocampal atrophy of the cognitive, social and physical sub-aggregates. Here, a hierarchical linear regression was conducted, which revealed significant negative correlations between bilateral hippocampal atrophy and cognitive activity (*r* = −0.53, *p* < 0.01), and between bilateral hippocampal atrophy and social activity (*r* = −0.47, *p* < 0.05) but no significant correlation between bilateral hippocampal atrophy and physical activity.

## Discussion

Consistent with our prediction, there was a significant negative association between EE (i.e., engagement in cognitive, physical, and social activities) at 5 months post-injury and bilateral hippocampal atrophy from 5 to 28 months post-injury. Although the observational design of the present study precludes conclusions regarding causality, our findings are consistent with previous associations found between EE and enhanced hippocampal structure, and they are potentially explained by increased production, survival and integration of newly generated dentate gyrus neurons (Kempermann et al., 2002; Olson et al., 2006; Tanti et al., 2013); findings in animals—where EE has been correlated with neurogenesis in TBI and moreover correlated with better recovery (Kovesdi et al., 2011; Matter et al., 2011)—bolster such an interpretation. At a behavioral level, the current findings are consistent with use-it-or-lose-it frameworks, and support the extrapolation of the Mahncke et al. (2006) framework—where reduced schedules of activities and avoidance of cognitively-challenging activities were purported to lead to under-stimulation of critical neural networks and loss of associated functions—to chronic TBI (Evans et al., 2008). The findings are also consistent with Robertson and Murre's (1999) computational model of brain plasticity and guided recovery of function, whereby damaged, but potentially viable circuits may be repaired via cognitive activity, cognitive arousal, and Hebbian learning mechanisms. As well, these findings converge with findings from the cognitive-training literature, which shows evidence that engagement in cognitively-demanding tasks is associated with increased cerebral volume in brain areas that are functionally-related to the demands of the task (Draganski et al., 2006).

Unexpectedly, the results of the present study showed no significant relationship between hippocampal atrophy and pre-injury cognitive reserve, as measured by level of education. This finding was surprising given the extensive evidence of a positive association between level of education and cognitive functioning in normal aging (Corral et al., 2006; Fritsch et al., 2007), stroke recovery (Elkins et al., 2006), and TBI recovery (Kesler et al., 2003). Therefore, the study findings do not offer evidence that this proxy for pre-injury EE protects against the structural progression of subacute hippocampal atrophy following TBI. One interpretation of this null finding is that a fixed degree of neural reserve at the time of brain injury does not confer neuroprotection against progressive pathology (e.g., disconnection, [Tate and Bigler, 2000]; neuroinflammation [Bigler, 2013; Johnson et al., 2013]) as would be logically predicted by conventional theories of brain reserve (Satz, 1993; Stern, 2002) and by negative associations found between premorbid cognitive reserve and progression of pathology in Alzheimers disease (Stern, 2006), multiple sclerosis (Amato et al., 2013) and stroke (Willis and Hakim, 2013). In particular, our findings suggest that in order for EE to positively modulate brain disorders via neuroprotective and/or compensatory mechanisms, EE exposure must occur after the disorder has commenced, and during the period of progression of neuropathology (Nithianantharajah and Hannan, 2009). Such an explanation is consistent with EE influences on neurogenesis, as discussed above (Kovesdi et al., 2011; Matter et al., 2011). As neurogenesis is an active process, it may be that temporally congruent factors, namely current EE, but not past education, play a more significant role (Lee et al., 2009; Surget et al., 2011). Unfortunately, our retrospective analysis did not allow us to quantitate the dentate gyrus, the site of neurogenesis.

EE in humans is viewed conventionally as being comprised of three primary elements: cognitive, physical, and social stimulation (Scarmeas and Stern, 2003; Studenski et al., 2006). Of the three conventional elements of EE, our preliminary findings indicated that cognitive activity accounted for the most outcome variance in bilateral hippocampal atrophy. The question whether it is cognitive, social or physical enrichment that confers the greatest neuroplastic advantage is under active debate; our findings are consistent with those comparison studies in animals that found greater survival and integration of new hippocampal neurons after cognitive stimulation than exercise (Kempermann et al., 2002, 2010; Olson et al., 2006; Curlik and Shors, 2011; Shors et al., 2012; Tanti et al., 2013).

A limitation of the present study was the small sample size and relatively small (albeit typical) representation of female subjects, which limits the generalizability of the study findings and precluded examination of gender as a control variable.

It is important to note that in our preliminary and exploratory analysis of the relative contributions of the sub-aggregates (cognitive vs. social vs. physical stimulation), the greater number of cognitive items on the LAQ may have conferred greater analytic stability to the cognitive outcome measure, with the very small number of physical items (2) providing limited stability. Therefore, further research into the relationship between cognitive vs. other types of stimulation and atrophy in the chronic stages of TBI is needed.

Many studies of EE in humans are observational and correlational in design, including the present study. Future EE research would benefit from inclusion of elements that would permit conclusions regarding causality and directionality of relationships, as offered by Salthouse et al. (2002). They suggested that random assignment to experimental and control groups would minimize influences of relevant pre-existing individual differences, such as initial level of cognitive ability and amount of education. Further, rigorous control of enrichment groups, in terms of type and amount of EE, is needed as well as long-term objective monitoring of the amount and frequency of EE activities. Though, given that objective monitoring of lifestyle activities is often unfeasible, experience sampling might offer greater accuracy than retrospective self-report (Csikszentmihalyi and Larson, 1987). Further general challenges in the measurement and manipulation of EE in humans pertain to inter-individual differences in motivation, interest and engagement: what is cognitively challenging, stimulating, and enjoyable to one may be overly difficult and thus stressful for another, or excessively easy and thus insufficiently stimulating to confer neural benefits to another. These limitations affect the strength of conclusions in our own study and warrant future experimental studies with the controls described above.

With regard to models of rehabilitation, the present study arguably provides preliminary empirical support for the contextualized model of cognitive rehabilitation proposed by Ylvisaker et al. (2002), in which therapeutic activities embedded in the patient's real life outside of the clinical setting may lead to greater recovery following brain injury. Cognitive rehabilitation researchers have recommended that rehabilitation strategies should be embedded in real-life contexts in order to maximize far transfer of learned skills and to impact real-life functioning (Murre and Robertson, 1999; Mateer and Sira, 2006). Consistent with these recommendations and with the Ylvisaker et al. (2002) contextualized model of rehabilitation, the majority of activities examined in the present study were embedded in real-life contexts, thus increasing the practical utility of the present study findings. Future prospective research should compare augmentation or intensification of those activities that are a part of patients' day to day life, with EE conferred by more conventional interventions such as computerized cognitively stimulating activities. Longitudinal measures of EE are also needed. Here, we inferred that EE at 5 months post-injury is a proxy for ongoing EE.

Regarding future research avenues, preliminary findings indicate that the default mode network (DMN; Raichle et al., 2001) in humans is implicated in TBI, with functional connectivity decrements within the DMN predicting sustained attention deficits (Bonnelle et al., 2011; Sandrone and Bacigaluppi, 2012). Future studies could investigate the benefits of EE factors purported to impact connectivity within the DMN, such as meditation practice (Taylor et al., 2013), an area of burgeoning interest in the literature on recovery from TBI.

## Conclusions

The present study is the first to examine EE factors and their relationship with hippocampal atrophy in the chronic stages of moderate-severe TBI. EE that followed TBI and was temporally proximal to injury had a negative association with hippocampal atrophy, while years of education, a proxy for pre-injury EE (or cognitive reserve) was not significantly associated. Validation of the present findings through replication with larger samples of TBI patients is needed, ideally in a study in which the type and duration of EE is experimentally manipulated. EE is intimately associated with modification of existing synapses, synaptogenesis as well as neurogenesis. Its role in neurorehabilitation appears critical. The findings of this correlational study can be used to generate testable hypotheses regarding the directional impact of EE, as well as the active ingredients of EE for buffering against cerebral atrophy, and its functional consequences. Such questions have significant implications for the development of effective rehabilitation methods for people suffering the long-term consequences of moderate-severe TBI.

### Conflict of interest statement

The authors declare that the research was conducted in the absence of any commercial or financial relationships that could be construed as a potential conflict of interest.
